# Unusual cause of unilateral facial oedema: intramuscular dirofilariasis

**DOI:** 10.1007/s00405-023-08057-y

**Published:** 2023-06-13

**Authors:** Deviprasad Dosemane, Meera Niranjan Khadilkar, Susmita Sriperumbudur, Ishaan Aggarwal

**Affiliations:** Department of Otorhinolaryngology and Head & Neck Surgery, Kasturba Medical College, Mangalore, Manipal Academy of Higher Education, Manipal, 575001 India

**Keywords:** Dirofilaria, Dirofilaria repens, Health, Parasitic infections, Parotid diseases

## Abstract

**Introduction:**

Unilateral facial oedema may occur due to inflammatory, infective, or cystic pathology; patients often seek medical help at an early stage.

**Case report:**

We report one such case caused mimicking a parotid abscess, caused by dirofilariasis.

**Conclusion:**

Dirofilariasis is an emerging zoonosis and should be considered a differential diagnosis of atypical facial swelling. It is equally important for clinicians, radiologists, and pathologists to be familiar with the diagnostic characteristics to avoid misdiagnosis.

## Introduction

Unilateral facial oedema may occur due to inflammatory, infective, or cystic pathology; patients often seek medical help at an early stage. Clinical history and examination, aided by imaging studies, help make a conclusive diagnosis. The most frequent causes of acute facial oedema are dental infections, sialadenitis, and orbital cellulitis; less common aetiology includes acute invasive fungal sinusitis, cavernous sinus thrombosis, and ranula and branchial cleft cysts [1]. We present an unusual case of unilateral facial oedema in a middle-aged patient.

## Case report

A middle-aged woman presented with pain and swelling on the right side of the cheek for 7 days. The patient gave a history of similar swellings over both eyelids and the left side of the face in the previous year. Examination revealed a diffuse, tender swelling with a smooth surface causing mild facial asymmetry. There was no fixity to the skin or underlying tissues. Ultrasonogram (USG) showed an early forming parotid abscess. Oral antibiotic therapy was initiated. The swelling subsided 1 week later; surprisingly, the repeat USG showed the presence of an ill-defined hypoechoic lesion of size 1.9*0.8*2.2 cm in the intramuscular plane involving the right masseter with a thin linear parallel tubular structure which was coiled and folded on itself likely to be a filarial parasite. The patient was planned for surgical removal of the parasite.

**Fig. 1:**
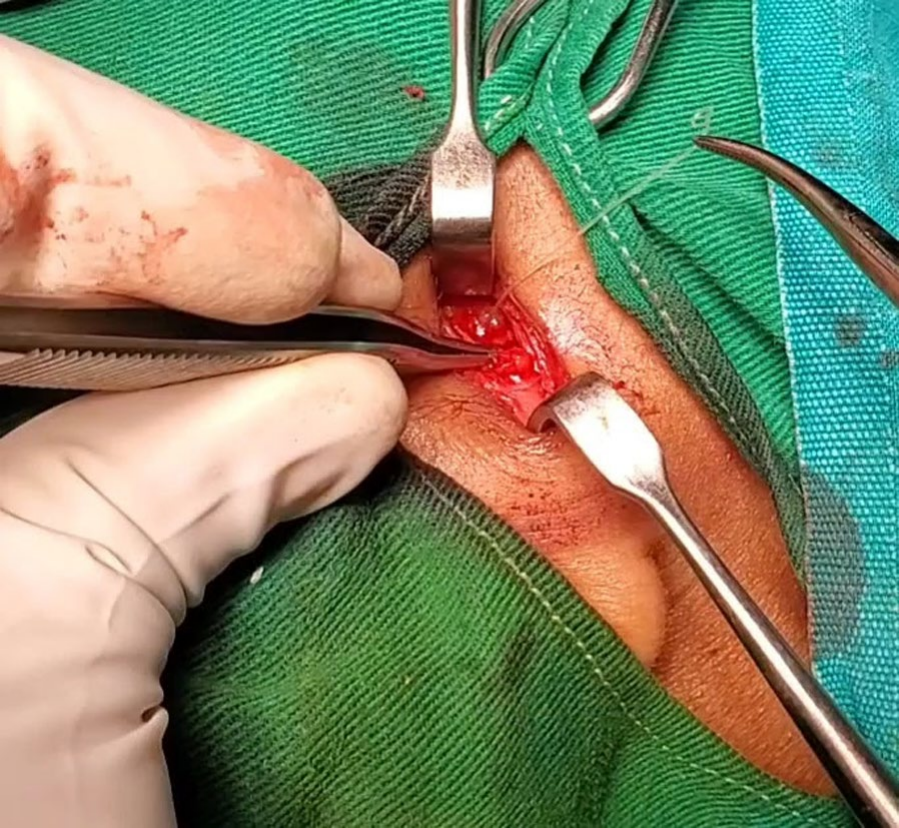
Intraoperative photograph of live dirofilarial worm extracted from right masseter

Under general anaesthesia, an incision was made on the right cheek 2 cm above the body of the mandible. On blunt soft-tissue dissection, a live worm was found in between the muscle fibres in the right masseter (Fig. 1). The parasite was removed and sent for microbiology examination, which confirmed Dirofilaria repens, a type of filarial worm. Postoperative recovery was uneventful.

## Discussion

Dirofilariasis is a zoonotic parasitic infection caused by filarial nematodes belonging to the genus Dirofilaria. The disease is typically found in animals, particularly canines, but accidental human infection can occur [2]. Dirofilarial nematodes are transmitted to humans through the bite of infected mosquitoes. Once infected, the larvae circulate in the bloodstream and migrate to various sites, including the subcutaneous tissue, where they develop into adult worms [3]. The prevalence of human dirofilariasis varies globally, with most cases reported in the Mediterranean region, Russia, and Asia; the prevalence in south India is about 4.4%, and most of the cases affect adults aged 41–60, while a few cases have been reported in northern Indian states of Uttar Pradesh, Haryana, and Punjab [4]. However, this was the first ever case we encountered in our practice.

Symptoms of infestation include swelling, inflammation, and pain in the affected area, typically limited to the skin and subcutaneous tissues with the development of nodules and severe cases may result in lung and heart damage [2, 5]. However, as reported in this case, the clinical presentation can be diverse and mimic other conditions, such as a parotid abscess. Das reported a case of paramassetric subcutaneous dirofilariasis which was surgically excised [6]; the present case had intramassetric presentation. Diagnosis poses a challenge due to the non-specific clinical presentation and the low sensitivity of serological tests, i.e., detection of antibodies to *D. immitis *[7]. Imaging techniques, such as ultrasound and MRI, and histological examination help in diagnosis [3].

Although uncommon, dirofilariasis should be considered in the differential diagnosis of facial swellings and subcutaneous nodules, particularly in individuals with a history of travel to or residence in endemic areas. Our patient most probably got infested with the worm from contact with her infected pet dog.

Early diagnosis and appropriate treatment can lead to successful outcomes in affected individuals. In humans, surgical removal of the worms is typically the treatment of choice, although antihelminthic drugs, such as diethylcarbamazine and ivermectin, may also be used [3]. In the present case, the patient had a history of recurrent swellings over the last year involving the left cheek and left eye. For every episode, she received symptomatic treatment and the swelling subsided. When she presented to us, the swelling mimicked a parotid abscess and was treated for the same. USG showed the presence of a parasite which was removed surgically.

Preventive measures for dirofilariasis include mosquito control, insect repellents, and proper disposal of waste to prevent mosquito breeding sites. Additionally, regular veterinary care and treatment of infected dogs can help reduce the risk of transmission to humans [4].

## Conclusion

The presented case highlights the importance of considering parasitic infections as a differential diagnosis in patients with swelling and pain in the facial region. Diagnosing dirofilariasis can be challenging, but early diagnosis and prompt treatment have improved outcomes. Awareness of the disease and its epidemiology in endemic areas are crucial in management. Further studies are needed to determine the prevalence, particularly in endemic areas.

## Data Availability

Data transparent.
